# ALK-positive diffuse large B-cell lymphoma of the duodenum: A case report and review of the literature

**DOI:** 10.3892/etm.2014.1786

**Published:** 2014-06-16

**Authors:** XIAOMING XING, DONGLIANG LIN, WENWEN RAN, HUAMIN LIU

**Affiliations:** 1 Department of Pathology, The Affiliated Hospital of Qingdao University Medical College, Qingdao, Shandong 266003, P.R. China; 2 Department of Oncology, The Affiliated Hospital of Qingdao University Medical College, Qingdao, Shandong 266003, P.R. China

**Keywords:** anaplastic lymphoma kinase, diffuse large B-cell lymphoma, duodenum

## Abstract

Anaplastic lymphoma kinase (ALK)-positive diffuse large B-cell lymphoma (ALK^+^ DLBCL) is characterized by the presence of immunoblastic or plasmablastic cells with a strong ALK protein expression that is frequently associated with t(2;17)(p23;q23). The present study reports a case of ALK^+^ DLBCL in a 26-year-old male with a duodenal mass. Histologically, the neoplastic cells demonstrated prominent plasmablastic differentiation with abundant amphophilic cytoplasma and central nucleoli. Paraffin immunohistochemistry revealed: an exclusively cytoplasmic granular expression of ALK; CD138, immunoglobulin A (IgA) and CD79α positivity; and focal expression of multiple myeloma oncogene 1 (Mum-1), CD30 and epithelial membrane antigen (EMA). However, the immunohistochemical staining was negative for CD3, CD38 and CD20. Fluorescence *in situ* hybridization (FISH) analysis using an ALK break-apart probe revealed the presence of ALK gene rearrangements in the patient. To the best of our knowledge, the current case represents the first example of primary extranodal ALK^+^ DLBCL presenting as a duodenal mass.

## Introduction

Anaplastic lymphoma kinase (ALK)-positive diffuse large B-cell lymphoma (ALK^+^ DLBCL) is a rare novel subtype of DLBCL that was recognized as a separate entity in the 2008 World Health Organization (WHO) classification of lymphoid neoplasms (1). Since it was first described in 1997 (2), to the best of our knowledge, ~60 cases have been reported to date. Morphologically, the lymphoma is composed of immunoblastic or plasmablastic cells and exhibits a sinusoidal growth pattern. Immunohistochemical staining of the tumor cells reveals a distinct profile, including a lack of B-lineage (CD20 and CD79α) and T-lineage (CD3) markers and CD30, but the expression of CD138 and CD38 (plasmacytic markers), variable expression levels of CD4 and CD57, and single light-chain cytoplasmic immunoglobulin A (IgA). Notably, a previous study demonstrated that lymphoma cells were strongly positive for ALK in a cytoplasmic granular staining pattern, which was different to the cytoplasmic and/or nuclear pattern characteristics of the T/null anaplastic large cell lymphoma (ALCL) (3).

Initially, an ALK gene rearrangement was not detected in ALK^+^ DLBCL and the full-length ALK protein was considered to be the pathogenesis of the lymphoma (2). However in 2003, Gascoyne *et al* (4) and De Paepe *et al* (5) described six and three cases, respectively, of ALK^+^ DLBCL. They were characterized by t(2;17)(p23;q23), which results in the fusion of the ALK gene at chromosome band 2p23 and the clathrin gene (CLTC) at 17q2. Subsequent studies revealed chromosome translocation at t(2;5)(p23;q35), which was frequently associated with ALCL, and a cryptic insertion of the ALK gene into chromosome 4 at band 4q22-24 fusion in certain cases of ALK^+^ DLBCL (6–8).

Although extranodal sites, including the nasopharynx and stomach, may be involved (7,9,10), it is the lymph nodes that are consistently primarily involved in cases of ALK^+^ DLBCL. The present study reports, to the best of our knowledge, the first case of primary extranodal ALK^+^ DLBCL presenting as a duodenal mass.

## Case report

### Case summary

A 26-year-old male presented with a principal complaint of abdominal distension and vomiting for almost three weeks. Computed tomography (CT) scans revealed an irregular mass in the lower part of the duodenum, abdominal and retroperitoneal lymphadenopathy, and multiple low-density foci in the spleen. However, there was no evidence of disease elsewhere. The endoscopic biopsy was reported as lymphoma, although a definitive diagnosis was not reached. A subsequent palliative pancreaticoduodenectomy was performed.

**Figure 1:**
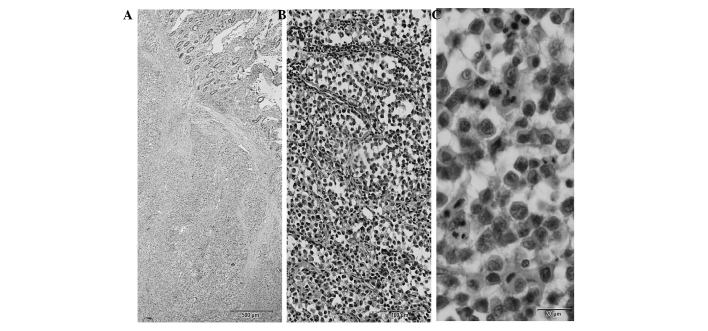
Cytologic features of the anaplastic lymphoma kinase-positive diffuse large B-cell lymphoma (ALK^+^ DLBCL). (A) Diffuse infiltration of tumor cells (HE stain; magnification, HE ×20). (B) Sinusoidal infiltration pattern (HE stain. magnification, HE ×100). (C) Tumor cells with round, regular nuclei; single, central, eosinophilic nucleoli and moderate amounts of eosinophilic cytoplasm (HE stain; magnification, HE ×400). HE, hematoxylin and eosin.

Histological examination of the tumor revealed a diffuse infiltration of tumor cells from the mucosa to the serosa. The neoplastic cells were large, with centrally or eccentrically located round nuclei, prominent single nucleoli and moderately eosinophilic cytoplasm (Fig. 1). Regional lymph nodes were also invaded. Bone marrow biopsy revealed no evidence of infiltrations by the lymphoma. The patient was assessed as being at stage IIa according to the Ann Arbor staging system.

Eight cycles of cyclophosphamide, doxorubicin, vincristine, prednisone and etoposide (CHOPE) were administered to the patient over nine months. The patient responded to the therapy and the disease was partially regressed, although without the achievement of tumor-free status.

The use of human tissue samples for this study was approved by the Institutional Review Board of the Affiliated Hospital of Qingdao University Medical College (Qingdao, China). The patient provided written informed consent for their participation in the study.

### Immunohistochemistry

Immunohistochemical staining was performed on paraffin-embedded tissue sections (5 μm) with the EnVision method. The analyses were conducted with a large panel of monoclonal antibodies, including antibodies against CD20, CD79α (both from ZSGB-BIO, Beijng, China), CD3 (BD Biosciences, Heidelberg, Germany), CD138, CD38, CD56, epithelial membrane antigen (EMA), AE1/AE3, CD30, multiple myeloma oncogene 1 (Mum-1), CD10 (all from ZSGB-BIO), B-cell lymphoma 6 (Bcl-6; BD Biosciences), immunoglobulin A (IgA; DAKO, Glostrup, Denmark) and Ki67 (ZSGB-BIO), following antigen retrieval. For detection of the ALK (BD Biosciences) protein, the monoclonal antibody anti-ALK was used.

### In situ hybridization

*In situ* hybridization was performed, according to standard methods (11), on 4-μm paraffin-embedded tissue sections with specific digoxigenin-labeled probes (ZSGB-BIO) that were complementary to Epstein-Barr virus (EBV)-encoded RNA nuclear transcripts.

### Fluorescence in situ hybridization (FISH)

FISH was performed on 4 μm paraffin-embedded tissue sections following deparaffinization and digestion. The slides were washed in saline-sodium citrate (SSC) buffer, fixed in 10%-buffered formalin for 5 min, dehydrated in graded alcohol and allowed to air dry. Hybridization was performed using a dual-color break-apart rearrangement probe (ZSGB-BIO) for the ALK gene on chromosome 2. The probes were denatured by incubation at 78°C for 5 min in a humidified box, after which they were hybridized overnight at 42°C.

### Immunohistochemistry and in situ hybridization results

**Figure 2:**
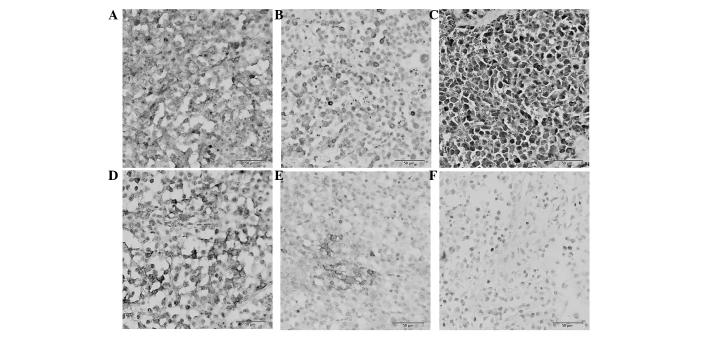
Typical immunohistochemical staining of the anaplastic lymphoma kinase-positive diffuse large B-cell lymphoma (ALK^+^ DLBCL). The tissue was positive for (A) CD138; (B) CD79α and (C) immunoglobulin A (IgA) and focally positive for (D) epithelial membrane antigen (EMA), (E) CD30 and (F) multiple myeloma oncogene 1 (Mum-1). Magnification, ×200 by the Envision method.

**Figure 3:**
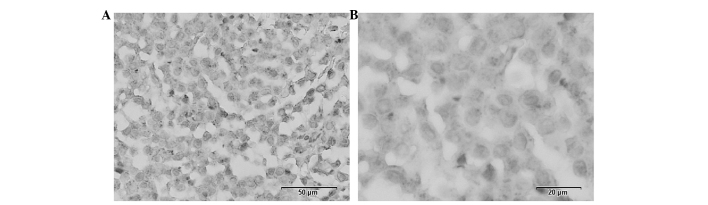
Granular cytoplasmic staining of the anaplastic lymphoma kinase (ALK). Magnification of (A) and (B) is ×200 and ×400, respectively by the Envision method.

Immunohistochemistry demonstrated that the tumor cells were positive for CD138, IgA and CD79α (Fig. 2), but negative for CD3, CD38, CD20, CD10, Bcl-6, CD56 and cytokeratin. Mum-1, CD30 and EMA revealed a patchy reactivity (Fig. 2). Furthermore, immunohistochemistry with the monoclonal antibody anti-ALK1, revealed a granular cytoplasmic expression of the ALK protein by neoplastic cells (Fig. 3). The EBV *in situ* hybridization staining was negative.

### FISH results indicate ALK gene rearrangements

**Figure 4:**
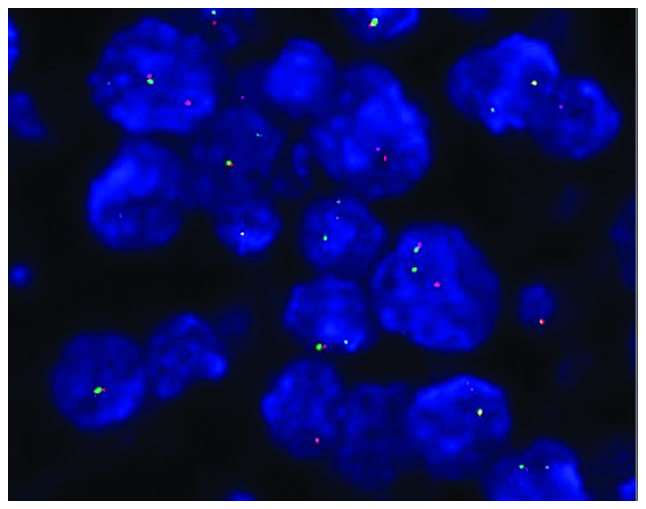
Fluorescence *in situ* hybridization of the anaplastic lymphoma kinase (ALK) gene using a break-apart probe. The clearly separated green and red signals indicate a translocation of the ALK gene.

FISH analysis demonstrated that gene rearrangement of ALK were present in the patient. Fig. 4 shows clearly separated green and red signals indicating the translocation of the ALK gene. The normal ALK gene signal is shown as a fused yellow signal or joined green and red signals.

## Discussion

ALK^+^ DLBCL was originally described by Delsol *et al* (2) in 1997, based on a series of seven cases. It spans all age groups (9–72 years), with a median of 38 years, and occurs with a male predominance (male:female ratio, 3:1). ALK^+^ DLBCL frequently presents with an aggressive clinical course, a poor response to therapy and a short survival time (12). Histologically, the tumor cells exhibit an immunoblastic and/or plasmablastic morphology with a sinusoidal growth pattern. Immunohistochemically, ALK^+^ DLBCL does not express B-lineage markers (CD20 and CD79α), T-lineage markers (CD3), cytotoxic granular proteins (granzyme B and TIA-1) or CD30. However, plasmacytic markers, including CD138, CD38 and EMA, are characteristically expressed, which demonstrates the terminally differentiated B-lineage origin of the tumor cells (13). In the majority of reported cases of ALK^+^ DLBCL, monotypic IgA λ was expressed, and EBV was not detected by *in situ* hybridization (3,5,6,9,10,13,14). In concordance with the results of these studies, the present case tested positive for the expression of CD138 and IgA.

Although a unique immunophenotypic profile has been established for ALK^+^ DLBCL, there remains a certain immunophenotypic heterogeneity. For example, CD79α is not usually expressed in tumor cells; however, in certain cases, including the case reported in the present study, the tumor cells were positive for CD79α (10,14). Similarly, although usually negative, the current case and one case described in the study by De Paepe *et al* (5) were focally positive for CD30.

Lymph nodes are consistently primarily involved in ALK^+^ DLBCL; however, cases of extranodal involvement, including in the stomach, nasal cavity, ovary and brain, have been described (7,9,10,14). While the gastrointestinal tract (GI) is the most common site of extranodal lymphoma, only one case of an extranodal lymphoma in the stomach has been reported, which was in the study by McManus *et al* (9). The study described a gastric ALK^+^ DLBCL without the involvement of bone marrow or adjacent organs in a patient aged 21 years (9). To the best of our knowledge, the current study describes the first case of ALK^+^ DLBCL in the duodenum, which represents the second reported occurrence of ALK^+^ DLBCL in the GI tract.

ALK^+^ DLBCL exhibits a distinct staining pattern for the ALK protein according to the underlying gene rearrangement. The majority of reported cases with clathrin heavy-chain (CLTC)-ALK gene translocation exhibit granular cytoplasmic staining (2,3,7,13,14), while a number of cases with nucleophosmin (NPM1)-ALK translocation exhibit cytoplasmic and nuclear staining (6,7). Furthermore, certain other staining patterns have been observed. Lee *et al* (13) observed a case demonstrating a unique nuclear membrane-staining pattern for the ALK protein, which indicated a novel ALK gene rearrangement involving another translocation partner gene. A cryptic 3′-ALK gene insertion into chromosome 4 was identified by Stachurski *et al* (8), which represented a novel cytogenetic alteration of ALK^+^ DLBCL. The current case demonstrated a typical cytoplasmic and granular ALK staining pattern and the corresponding rearrangement of the ALK gene.

Clinically, ALK^+^ DLBCL is more aggressive and reveals a worse response rate to standard chemotherapy compared with typical DLBCL. Laurent *et al* (14) reviewed a large cohort of 38 cases of ALK^+^ DLBCL and observed that the majority of patients experienced an aggressive clinical course, with a 5-year survival rate of 25% following treatment with the CHOPE regimen. Since tests for CD20 are negative in the majority of cases of ALK^+^ DLBCL, rituximab plays no therapeutic role. Therefore, it is important to develop novel and effective alternative treatments for ALK^+^ DLBCL, including front-line intensification with or without autologous stem-cell transplantation, and the application of new biological agents such as anti-CD138 monoclonal antibodies and ALK inhibitors (14,15).
